# Experiments on the Effects of Apertures Made into the Chest

**Published:** 1823-06

**Authors:** David Williams


### ART. VIII.—*Experiments on the Effects of Apertures made into the Chest.* By DAVID WILLIAMS, M.D.

THE following experiments were instituted from a desire to examine a very important physiological hypothesis, advanced by Dr. CARSON. That gentleman has published a pamphlet,* consisting of three essays,—one, on the Elasticity of the Lungs; another, on the Vacuity of the Arteries after Death; and the third, on Lesions of the Lungs. In the second essay, or that on the Vacuity of the Arteries after Death, he relates two methods that he adopted for killing animals, by admitting air through different parts of the parietes of the chest into both its cavities simultaneously, thereby collapsing the lungs. One method was by making an opening, of about an inch in length, between a pair of the ribs on each side of the thorax. The

* *Essays, Physiological and Practical.* By JAMES CARSON, M.D. Liverpool, 1823.

other was by making an opening, fit to admit his two fingers, from within the abdomen, through the muscular part of the diaphragm on each side. Instantaneous death was the result of the latter method in all his trials. In the former, death was not less certain, though a little more tedious: the life of one animal was protracted for twenty minutes. In his third essay, or that on Lesions of the Lungs, Dr. Carson details an experiment performed on three rabbits, as follows:—An incision was made between two of the ribs, on one side, into the cavity of the chest, and the air was freely admitted, (when he concluded that the lung must have been reduced to a state of collapse;) then the wound was allowed to close, and heal. At the distance of five days, a similar opening was made into the cavity of the opposite side; when one out of three rabbits soon expired after the second operation. Dr. Carson inferred from the result, that it is possible to collapse one of the lungs, and to retain it in that state *ad libitum*, by keeping open the communication between the cavity of the chest and the external air; and, further, that, upon allowing the opening to close, the lung in a given time will resume its wonted function, thereby rendering it practicable, when conceived necessary, to place the opposite lung under the like discipline. Dr. Carson has very ingeniously proposed the application of his views to the cure of one of the most frequent and fatal of diseases, phthisis pulmonalis; and, in a single instance, he has reduced his hypothesis to practice, by sanctioning the operation on a consumptive patient in this town.

It will be, perhaps, rather difficult to reconcile with the above the results of the following experiments: indeed, had I not been indulged with the presence of medical gentlemen at several of them, I should have felt some scruple in giving them publicity. All the subjects of my experiments were dogs: the four first noticed had their os humeri secured to the rings of 56℔. weights; their hind legs were well extended, and fastened to similar weights; so that they were retained nearly in their natural standing position, which preserved to them, in a great measure, the use of their auxiliary respiratory powers.

1*st Experiment.*—An opening, to the full extent of an inch, was made into the left cavity of the chest, between the sixth and seventh ribs, mid space between the spine and sternum. At each inspiration, the lung was partially inflated, and was distinctly seen beneath the orifice, and at a little distance from it. At each expiration, it evidently contracted, and its lower thin margin was thrust outward through the aperture with great force and a peculiar noise, caused by the protrusion and an exit of a portion of the air. The breathing was distressed when the lung protruded; but, as soon as the lung was allowed to contract, it became more tranquil. The right cavity was in like manner opened, and the same phenomena were observed. When both apertures were kept wide open, the breathing was very laborious and distressing; which did not seem to arise altogether from the presence of air in the cavities, but principally from the violent protrusion of the inferior margins of the lungs; because the instant the openings were permitted to contract, so as to prevent that accident, respiration became more easy. About ten minutes elapsed after the second opening was made, before the lips of the wounds were brought together. The dog, when let loose, walked, was weak, but breathed easy; would taste nothing until the next morning, when he appeared lively, took his food, and regained his strength daily. The ninth day after the operation he was hanged, and the trachea tied, to prevent the lungs collapsing on opening the chest.

*Dissection.*—No morbid appearance in the cavities of the chest; the wounds were healed internally, but not externally. The lungs did not reach, by two inches, the lowermost part of their cavities; nor did their bulk appear equal to the filling of the remaining part of their cavities.

*2d Experiment.*—Dr. Traill, and Mr. Christian and Mr. Dawson, surgeons, present. The operation was performed in the same manner as the first. The openings were better than an inch in length. Both lungs were wounded in the operation, in consequence of the animal struggling, his hind legs not being sufficiently extended. The lungs did not protrude; but the wounded part of the one that was most seriously injured by the knife was observed to present itself, at each expiration, opposite the aperture. Will a wounded lung protrude under every circumstance that a sound one will? The above medical gentlemen were convinced that the lungs only partially contracted, and that they were not in a quiescent state; but that respiration more or less oppressed, according as the openings were expanded, continued uninterruptedly during the experiment. A silver catheter, introduced into the chest, was moved about by the alternate contraction and distention of the lung. The dog was detained, with both apertures wide open, for the space of five minutes; and then untied, without bringing the edges of the wounds together. He was lively, shook his tail, drew himself upon his belly along the floor; then lay down on one side, and contracted himself, as if to close the openings: whether it was instinct or accident that induced him to choose that position, it certainly had that effect, thereby enabling him to breathe easier. Though his breathing, after he was let loose, did not appear much oppressed, his strength was considerably exhausted. About an hour after, the edges of the wounds were brought together: during the intermediate hour, the external air communicated with the cavities. He refused his food until next day, when he recovered his appetite, and regained his strength apace. The fifth day after the operation, the dog was hanged.

*Dissection.*—The internal edges of the wounds healed; the left lung had a deep-coloured circular spot, as large as the circumference of a crown-piece, surrounding the wounded part, which was quite healed. On making an incision into the discoloured portion, it was found gorged with blood, without any apparent disorganization, and appeared to be in a fair way of regaining its natural state. A similar spot, surrounding the wounded part, was observed on the right lung, but smaller: that wound was also healed. No adhesion or extravasation in either cavity. Absorption must have been rapid, as a considerable effusion of blood into the left cavity had taken place during the operation.

*3d Experiment.*—In the presence of Drs. Jeffrey, Jardine, Nicholson, and Messrs. Blackburn and Jones. An opening, of an inch and a half in length, was made between the sixth and seventh ribs, on both sides of the thorax of a middle-sized cur. The air had free ingress during inspiration; and at each expiration it rushed out, the lung frequently protruding, when not prevented by the examinations of the above medical gentlemen. The breathing was much oppressed. The animal was kept on the table five minutes after both the openings were made: when let loose, he walked about, apparently but little affected, the air passing in and out of the cavities. At the expiration of ten minutes from the time he was liberated, the apertures were fully distended, and retained so by applying and pressing a finger at the extremity of each of them, which prevented the ribs from approaching each other: in fact, in my opinion, they were fixed. The death of the animal took place in less than two minutes. To what extent the auxiliary respiratory organs were impeded in their action by the two ribs on each side being retained asunder, I cannot say; but, if they were impeded, and I think it is evident that they were, the death of the animal naturally must have been accelerated.

*4th Experiment.*—In the presence of the above gentlemen. A small opening was made into each cavity of the chest, in the intercostal spaces, of a bull-dog, and a full-sized clyster-pipe introduced into both apertures, which were retained for half an hour in an oblique position, their internal ends pointing towards the upper part of the thorax; during which time the air freely passed inwards and outwards. The animal was occasionally disturbed in his breathing; when he would struggle and contract, so as to force out, as much as possible, the air in the bags of the pleuræ; then he would make a quick and full inspiration, by which he was considerably relieved. After regaining his liberty, he seemed but little injured by the operation.

*5th Experiment.*—An opening, five inches in length, was made between the sternum and symphysis pubis into the cavity of the abdomen. The diaphragm appeared tense, and the action of its fibres were visible, but confined, from the very great pressure required to prevent the protrusion of the intestines, &c. The violent thrusting outward of the contents of the abdomen was principally, if not altogether, occasioned by the action of the diaphragm; for the force of the protrusion diminished as soon as its action on one side was obstructed, and ceased when it took place on both sides. With a little difficulty, an opening, about an inch in length, was made into the left cavity of the chest, through the muscular part of the diaphragm, three inches below its attachment to the ribs. When the air rushed in, the diaphragm on that side became relaxed, and its action not only obstructed, but its irritability to all appearance suspended; a portion of the diaphragm jutted out towards the abdomen, and formed a pouch, with the aperture in its centre. A similar opening was made into the right cavity of the chest, followed by the same effects. An ivory tube, three-tenths of an inch in diameter, was repeatedly introduced into each aperture. The diaphragm seemed to be altogether guided by the action of the contiguous parts, and by the pressure and passage of the air, modified by respiration: it was drawn towards the chest, and expanded by the dilatation of the thorax; but its expansion was not sufficient to obliterate the pouches before noticed, which was a strong proof, together with the quiescent state, compared with the previous violent thrusting outward of the contents of the abdomen, of its paralysed condition. Is it not likely that, in many wounds penetrating into the cavity of the abdomen, unaccompanied with thrusting outwards of its contents, that the diaphragm is wounded? At each expiration, it was pushed towards the abdomen, by the pressure and passage of the air from within the cavities of the chest, and the pouches enlarged. The breathing was oppressed, but not so distressing as in the three first experiments, when the apertures were wide open. The external wound was brought together by suture. When the dog was removed from the table, he walked, but tottered a little; he breathed easy; and three hours after lapped some milk, which was rather surprising, as the two first operated upon would accept of nothing until the following morning. He recovered his strength and liveliness amazingly fast. On the third day after the operation he was hanged, together with another dog not operated upon, for the purpose of comparing the appearances of their respective lungs: each had his trachea secured immediately on being cut down, before the suspending cord was slackened, to prevent the collapse of the lungs on opening the cavity of the chest.

*Dissection.*—In the presence of Dr. Traill, to whom I am greatly indebted for many suggestions in conducting these experiments. No morbid appearance in the abdomen, with the exception of a few adhesions between the liver and peritoneum. The apertures in the diaphragm were closed by a tender film, which very readily gave way to the probe. On opening the chest, the heart was observed once to contract; and the pericardium was perfectly transparent, as remarked by Professor Richerand. We could perceive very distinctly the ramifications of the coronary vessels. The diaphragm was in its natural position; no adhesions. The lungs, on first exposing of them, were perfectly smooth and glossy; in a little time they were slightly corrugated, by the effect of the cold in condensing the enclosed air: the examination was conducted in the open air. Not the slightest difference could be perceived, either in the expansion or general appearance of the lungs of the two dogs. Their lungs did not extend, by two inches, to the inferior part of their respective cavities; and their bulk appeared insufficient to fill the remaining part of the bags of the pleuræ.—Pray, what proof is there that the lungs fill the bags of the pleuræ? I must confess that I am quite sceptical upon that point; and, on the contrary, believe that in a healthy state they never completely fill them. To prove that the measure we had recourse to was effectual in preventing the air escaping out of the lungs when the chest was opened, at the conclusion, we divided the windpipe of each below the knots, when they instantaneously collapsed.

*6th Experiment.*—The description supposes the animal to be standing upright on his hind legs. The right cavity of the chest was transfixed with a sharp-pointed penknife, by thrusting it transversely, with a slight degree of obliquity downwards, through the intercostal space, immediately above the upper edge of the eleventh rib, mid space between its head and anterior extremity. The animal was afterwards put to death.

*Dissection.*—The instrument had pierced the diaphragm, and had slightly wounded the liver; the lung uninjured; the knife had passed as near as possible through the centre of the cavity at that part, and but little short of two inches above its inferior termination. Had the lung extended so low down it must have been wounded, the knife being sharp-pointed, and pushed in with force.

*7th Experiment.*—To remove every doubt with respect to the state of the lung when deprived entirely of the influence of the auxiliary respiratory powers, the following experiment was made in the open air: The cartilages of all the true ribs were divided, with the exception of the superior one, close to their juncture with the osseous structure, and the left cavity of the chest laid open its whole length; the diaphragm was punctured; the ribs, and their cartilaginous ends attached to the sternum, were separated and retained asunder, so as to expose and to deprive the lung of every assistance from the auxiliary respiratory organs. The lung rested upon the ribs and side of the vertebræ. On exposure, it shrunk considerably, but did not collapse. Its motion (for it was not at rest,) might be compared to that of a leech, when it draws up and again recedes its body without making progression; indeed, with both ends fixed: the motion was not that of pulsation, neither was it synchronous with the action of the heart; but it was a slow, undulating movement. On closing the gash, and keeping it so for four or five inspirations, then opening it quickly, the lung was found to have increased in bulk: on exposure, it again diminished. To close the scene, the knife was plunged into the heart, with a determination never to perform another experiment on a living animal; to which I was induced by my anxiety to solve an important practical problem.

I believe no one has ever appreciated the powers of the lungs and their auxiliaries in respiration during life, in resisting and diminishing the pressure of the atmosphere, when admitted into the cavities of the thorax through apertures in their parietes. That they do possess a considerable power, is beyond a doubt. The oppression of the breathing, and the expansion of the lungs, are in an inverse ratio to one another, and can be regulated by adjusting the dimensions of the openings into the cavities of the chest. As the apertures increase in their size, the power of expansion of the lungs is diminished, while the oppression of the breathing is augmented, until at last life is extinguished. What quantity of air is sufficient to produce death, as long as all the respiratory organs are not restrained, I have not ascertained. The doctrine of one lung collapsing, while the function of the opposite one is unimpaired, on exposure of its external surface to the atmosphere, taught from time immemorial in the schools, I must now consider as erroneous, and feel somewhat surprised that a notion so groundless should have existed for so many ages.

From the foregoing experiments it appears—

1st. That a lung will not collapse from exposure to the atmosphere, as long as respiration is carried on by the opposite one.

2d. That a lung possesses for a time, independently of the influence of the diaphragm and intercostal muscles, if respiration is carried on by the opposite one, a peculiar motive power; the source of which I do not pretend to explain.

3d. That a sound lung soon regains its full power of expansion when the pressure of the exterior air is removed.

4th. That air, freely and uninterruptedly admitted into both the cavities of the chest simultaneously, through tubes of a certain calibre, will not collapse the lungs, if the auxiliary respiratory organs are unrestrained.

5th. That air admitted into both the cavities of the chest (of a middle-sized dog) simultaneously, through apertures of an inch and better in length, in the intercostal spaces, will not collapse the lungs, provided the animal is allowed, unconfined, the use of his respiratory organs.

6th. That a sound lung never fills the bag of the pleura, at least during ordinary respiration.

If the last physiological inference is correct, it is highly interesting in a pathological point of view. It enables us to explain how hydrops thoracis, or that species of it, hydrops pleuræ, may exist to a certain extent, without being attended with any symptoms indicating the presence of the disease, as related by numerous medical authors. We can also more satisfactorily account how the lung so frequently escapes being wounded, when weapons penetrate the cavity of the thorax; and how the extravasation which follows, if not considerable, produces but little derangement. It may also have a practical utility; for it informs the surgeon that the lung descends to a certain point only, so that he need not be afraid of wounding it, should an operation be required below that position.

N. B.—Dr. Carson retained open the apertures into the chests of the animals he operated on with his fingers, whereas mine were kept open in the manner described; which accounts for the different results of our experiments. Dr. C., in his essays, does not allude to this circumstance, but he has mentioned it since my investigations. When air is admitted into the cavities of the chest, the animal requires the aid of all his respiratory organs to carry on respiration.

